# 1480. Detection of Multiple HIV Strains in an HIV-positive Transplant Recipient from an Acutely HIV Infected Donor.

**DOI:** 10.1093/ofid/ofad500.1316

**Published:** 2023-11-27

**Authors:** Naseem Alavian, Tatianna Travieso, Maria Blasi, Cameron R Wolfe

**Affiliations:** 1Department of Medicine, Division of Infectious Diseases Duke University School of Medicine, Durham, North Carolina; Duke University School of Medicine, Division of Infectious Diseases, Duke Human Vaccine Institute, Durham, North Carolina; Duke University School of Medicine, Division of Infectious Diseases, Duke Human Vaccine Institute, Durham, North Carolina; Duke University, Durham, North Carolina

## Abstract

**Background:**

The HIV Organ Policy Equity (HOPE) Act allows persons living with human immunodeficiency virus (HIV) infection to accept HIV-positive donor organs. Risk of superinfection or viral recombination resulting from the transmission of a genetically distinct donor HIV-1 strain may be a concern. It is unknown if transplantation from acutely infected donors pose additional risk. Analysis of viral quasispecies from donor and recipient samples provides an opportunity to evaluate the transplanted kidney as a viral reservoir and explore the potential for viral superinfection or recombination in the recipient.

**Methods:**

A 63-year-old HIV-positive recipient with focal segmental glomerulosclerosis and suppressed viral load (< 20 copies/mL) on antiretroviral therapy (ART) with dolutegravir and lamivudine, received a kidney from an acutely infected deceased HIV-positive donor **(Table 1).** Single genome amplification of the HIV-1 *env* gene was performed with viral RNA extracted from recipient urine and plasma and viral DNA from donor kidney cells, recipient PBMC and renal cells. We constructed phylogenetic trees assessed recombination probabilities.

**Figure:**
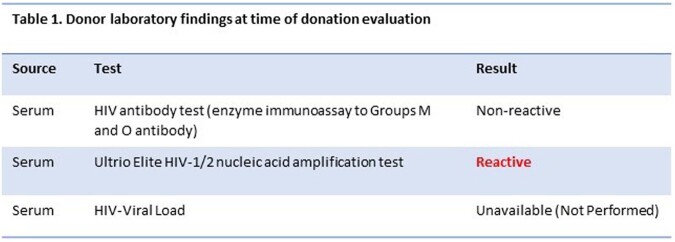

**Results:**

A new, genetically distinct *env* sequence was amplified from the recipient’s urine 30 hours post-transplant suggesting transfer of donor virus to recipient. Donor virus became undetectable on subsequent visits while recipient was maintained on ART. Notably, a third virus was detected 1.5 months post-transplant in urine-derived renal tubular epithelial cells and 5 months post-transplant in urine which was genetically distinct from previously detected donor and recipient strains **(Figure 1)**. This third strain did not have genetic features suggestive of recombination. Our findings suggest potential superinfection in the donor or recipient.

**Figure 1.**
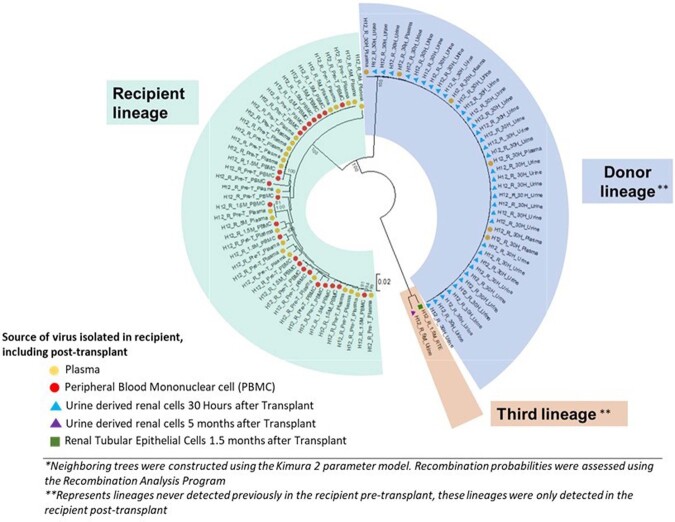
Phylogenetic Analysis of HIV Quasispecies Isolated from an HIV-Positive Kidney Transplant Recipient, Showing Three Distinct Viral Lineages*

**Conclusion:**

We documented presence of three unrelated HIV strains within a transplanted recipient and within different viral reservoirs. Donor and recipient recombination did not occur. In acutely infected donors, when the transfer of virus within the kidney occurs, ongoing virologic suppression can be achieved if appropriate ART is maintained. Additional long-term monitoring of viral populations in the recipient is important to fully assess any clinical and virologic implications of this finding.

**Disclosures:**

**All Authors**: No reported disclosures

